# Moringa oleifera supplementation by patients on antiretroviral therapy

**DOI:** 10.1186/1758-2652-13-S4-P188

**Published:** 2010-11-08

**Authors:** TG Monera, CC Maponga

**Affiliations:** 1 University of Zimbabwe College of Health Sciences, School of Pharmacy, Harare, Zimbabwe; 2 University of Zimbabwe College of Health Sciences, PO BOX A167,, Harare, Zimbabwe

## Purpose of the study

This survey determined the extent to which the herb Moringa oleifera commonly used for medicinal and nutritional purposes is being consumed among HIV positive patients.

## Methods

The study was a cross-sectional survey carried out at Parirenyatwa Hospital Opportunistic Infections Clinic. A convenience sample of 263 HIV-infected adults was taken from the Zimbabwe National Antiretroviral Roll-out Program. Using a previously piloted researcher administered questionnaire; patients who reported to the clinic over six months were interviewed about their use of herbal medicines. The focus was on Moringa oleifera use, and included plant part, dosage, prescribers and the associated medical conditions.

## Summary of results

Sixty-eight percent (68%) of the study participants consumed Moringa oleifera. Of these, 81% had already commenced antiretroviral drugs. Friends or relatives were the most common source of a recommendation for use of the herb (69%). Most (80%) consumed Moringa oleifera to boost the immune system. The leaf powder was mainly used, either alone (41%) or in combination with the root and/or bark (37%).

## Conclusions

Moringa oleifera supplementation is common among HIV positive people. Because it is frequently prescribed by non-professionals and taken concomitantly with conventional medicine, it poses a potential risk for herb-drug interactions. Patient medication history taking should probe for herbal supplementation and appropriate counselling done. Further experimental investigations into its effect on drug metabolism and transport would be useful in improving the clinical outcome of HIV positive patients on HAART.

